# Predeductible Coverage and Receipt of Telemental Health Visits

**DOI:** 10.1001/jamanetworkopen.2024.20731

**Published:** 2024-07-09

**Authors:** Kacey Fang, Jaclyn Marshall, A. Mark Fendrick, Haiden A. Huskamp, Latoya Thomas, Ateev Mehrotra

**Affiliations:** 1 Department of Health Care Policy, Harvard Medical School, Boston, Massachusetts; 2 Department of Health Economics and Outcomes Research, Included Health, Minneapolis, Minnesota; 3 Center for Value-Based Insurance Design, University of Michigan, Ann Arbor; 4 Department of Policy and Government Affairs, Included Health, Washington, DC; 5 Division of General Internal Medicine Beth Israel Deaconess Medical Center, Boston, Massachusetts

## Abstract

This cohort study examines the utilization changes associated with the reintroduction of cost sharing for patients receiving telemental health services.

## Introduction

The marked increase in telehealth visits, particularly for mental health, during the COVID-19 pandemic^1^ was bolstered by regulatory changes such as the exemption of telehealth visits from the deductible in high-deductible health plans, plans in which individuals face a minimum $1600 deductible.^2^ Congress extended this exemption through the end of 2024, and there is ongoing debate on whether the exemption should be made permanent.^3^ Given that there has been no empirical data on the effect of this exemption, we examined utilization changes associated with the reintroduction of cost sharing for patients receiving telemental health.

## Methods

The cohort study included patients from all 50 states and the District of Columbia receiving telemental health care from Included Health (a national telehealth-only company) from 2 clients (1 employer and 1 insurance plan) that varied in their coverage of telemental health during the study period. During the preintervention period (January 1 to June 30, 2021), all patients had no cost sharing for telehealth visits. In July 2021, one client (intervention) reintroduced cost sharing, and the other client (control) continued to offer telehealth services without cost sharing. The Harvard Longwood Campus institutional review board approved this study; informed consent was waived and the study was exempted from human participants review because of the use of deidentified data. This study followed the STROBE reporting guideline.

We used difference-in-difference methodology to evaluate how the reintroduction of cost sharing was associated with telehealth use in the postintervention period (July 1 to December 31, 2021). Among patients receiving care in the preintervention period, our 2 outcomes were the number of telemental health visits per patient and the proportion of patients who had any visits in the postintervention period. Models were adjusted for clinical and demographic characteristics, including a social deprivation index^4^ (eAppendix in Supplement 1). All *P* values were from 2-sided tests, and results were deemed statistically significant at *P* < .05. Analyses were performed using R, version 4.3.1.

## Results

There were 15 024 patients (6940 in intervention group; 8084 in control group) (Table). Across the entire cohort, the mean (SD) age was 33.5 (10.6) years with 71.4% of patients being female and 28.6% being male. Intervention cohort patients were more likely than those in the control cohort to live in urban areas (95.1% vs 79.0%; *P* < .001). In the 6-month preintervention period, the intervention and control groups had a mean (SD) of 4.8 (5.0) and 4.7 (5.0) visits per patient, respectively. In the postintervention period, the mean (SD) out-of-pocket costs per visit in the intervention and control groups were $29.50 ($30.00) and $0 ($0.08), respectively.

**Table.** Comparison of Patient Characteristics During the Preintervention Period, January to June 2021

Characteristic	Intervention (n = 6940)	Control (n = 8084)	*t* or χ^2^ Value	*P* value
No. of visits per patient, mean (SD)	4.8 (5.0)	4.7 (5.0)	0.76^a^	.45
No. of clinicians per patient, mean (SD)	1.4 (0.7)	1.4 (0.7)	−3.16^a^	.002
Age, mean (SD), y	34.3 (10.6)	32.8 (10.6)	8.63^a^	<.001
Sex, No. (%)				
Female	5118 (73.7)	5607 (69.4)	34.97^b^	<.001
Male	1790 (25.8)	2415 (29.9)	30.66^b^	<.001
PHQ-9, No. (%) in category^c^				
No to minimal depression (0-4)	2131 (30.7)	2089 (25.8)	43.51^b^	<.001
Mild depression (5-9)	1928 (27.8)	2114 (26.2)	4.97^b^	.03
Moderate to severe depression (≥10)	2278 (32.8)	3881 (48.0)	355.3^b^	<.001
Missing PHQ-9	603 (8.7)	438 (5.4)	61.44^b^	<.001
Social Deprivation Index score, mean (SD)	48.3 (28.4)	51.2 (25.3)	−6.70^a^	<.001
White patients, mean (SD), %^d^	73.5 (19.3)	77.8 (17.7)	−13.97^a^	<.001
Urban-rural status, rural, No. (%)^e^	341 (4.9)	1694 (21.0)	819.2^b^	<.001
No. of psychiatrists per 30 000 in county, mean (SD)	3.3 (2.5)	2.1 (2.2)	32.39^a^	<.001

Abbreviation: PHQ-9, 9-item Patient Health Questionnaire.

^a^ *t* Value.

^b^ χ^2^ Value.

^c^ First PHQ-9 captured from January to June 2021.

^d^ Measured at the zip code tabulation area by the US Census Bureau and underlying data not captured at the individual patient level.

^e^ Rurality is defined by the US Department of Agriculture’s Economic Research Service Rural-Urban Commuting Area; rural is defined as isolated, small rural, and large rural areas.

After reintroduction of cost sharing, the mean number of visits per patient per month was lower in the intervention group than the control group (Figure). In adjusted models, cost sharing was associated with 1.5 (95% CI 1.2-1.7; *P* < .001) fewer visits per patient and an 11.7% (95% CI, 10.1%-13.4%; *P* < .001) reduction in the proportion of patients who had any visits in the postintervention period (22.0% relative reduction).

**Figure.**
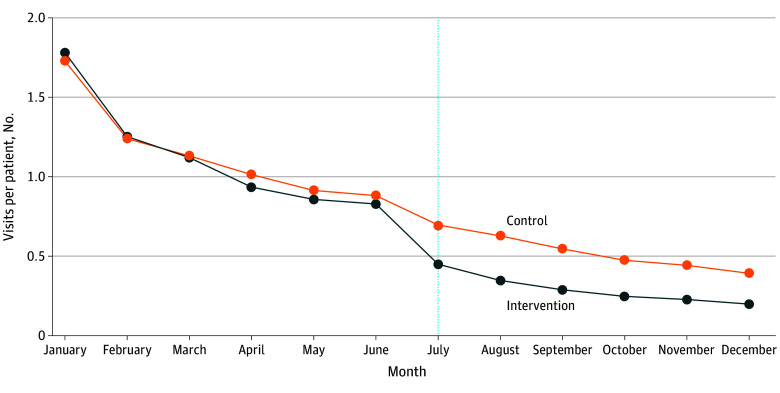
Number of Telemental Health Visits per Patient in Cohort, by Treatment Group Cost sharing for telehealth visits introduced in July 2021 (vertical dotted line) in the intervention group.

## Discussion

We found that when patients were required to pay out of pocket for telehealth visits, they had substantially fewer telemental health visits, and a larger fraction stopped seeing their mental health specialists. These findings imply that the expiration of the predeductible telehealth coverage exception in January 2025 may reduce mental health service use, which could lead to worse clinical outcomes. This study is limited by the sample being from a single telehealth company, the inability to determine whether patients received care from other clinicians, and a lack of data on clinical outcomes.

Our findings are consistent with a robust body of research showing that patient cost sharing decreases the use of both high- and low-value care.^5,6^ Given ongoing concerns about access to mental health treatment and to help patients stay in treatment, policies that reduce cost sharing for both in-person and telemental health visits could be considered.

## Notes

Supplement 1.
eAppendix.

eReference.

Supplement 2.
Data Sharing Statement
