# Supplementary material for: Predeductible Coverage and Receipt of Telemental Health Visits
Source: JAMA Netw Open. 2024 Jul 9;7(7):e2420731. doi: 10.1001/jamanetworkopen.2024.20731 (PMC11234231; doi:10.1001/jamanetworkopen.2024.20731)
Supplement: Supplement 1. — eAppendix. eReference. [file jamanetwopen-e2420731-s001.pdf]

Supplementary Online Content

Fang K, Marshall J, Fendrick AM, Huskamp HA, Thomas L, Mehrotra A. Predeductible coverage and receipt of telemental health visits. *JAMA Netw Open.* 2024;7(7):e2420731. doi:10.1001/jamanetworkopen.2024.20731

eAppendix.
eReference.

This supplementary material has been provided by the authors to give readers additional information about their work.

© 2024 Fang K et al. *JAMA Network Open.*

eAppendix.

Variables Used in multivariable linear models beyond intervention and time period variables. Sensitivity analyses using a Poisson distribution were similar.

Age – included as a continuous variable
Sex
Social Deprivation Index score – quintiles, with higher scores indicating greater social deprivation
ERS Rural-Urban Commuting Area code – 4 categories (isolated, small rural, large rural, urban)
Patient Health Questionnaire-9 score – 3 categories (0-4: no to minimal depression, 5-9: mild depression, 10+: moderate to severe depression)

Assumptions needed for using difference-in-difference methodology:

1. The assignment of the intervention is not influenced by the outcome.
2. In the absence of treatment, the intervention and control groups have parallel trends (i.e. the difference in the intervention and control groups would have been constant over time without the treatment).
3. The common shocks assumption is that any events after the policy changed equally impact both the intervention and control populations.

© 2024 Fang K et al. *JAMA Network Open.*

eReference.

Dimick, Justin B., and Andrew M. Ryan. "Methods for evaluating changes in health care policy: the difference-in-differences approach." JAMA 312.22 (2014): 2401-2402.

© 2024 Fang K et al. *JAMA Network Open.*
