# Supplementary material for: Predeductible Coverage and Receipt of Telemental Health Visits
Source: JAMA Netw Open. 2024 Jul 9;7(7):e2420731. doi: 10.1001/jamanetworkopen.2024.20731 (PMC11234231; doi:10.1001/jamanetworkopen.2024.20731)
Supplement: Supplement 2. — Data Sharing Statement [file jamanetwopen-e2420731-s002.pdf]

# Data Sharing Statement

Fang. Predeductible Coverage and Receipt of Telemental Health Visits. *JAMA Netw Open*. Published July 09, 2024. doi:10.1001/jamanetworkopen.2024.20731

**Data**
**Data available:** No

**Additional Information**
**Explanation for why data not available:** Our Data Use Agreement does not allow us to share the data.
